# Illuminating the immunological landscape: mitochondrial gene defects in pancreatic cancer through a multiomics lens

**DOI:** 10.3389/fimmu.2024.1375143

**Published:** 2024-03-06

**Authors:** Hao Chi, Lanqian Su, Yalan Yan, Xiang Gu, Ke Su, Han Li, Lili Yu, Jie Liu, Jue Wang, Qibiao Wu, Guanhu Yang

**Affiliations:** ^1^ Faculty of Chinese Medicine, and State Key Laboratory of Quality Research in Chinese Medicine, and University Hospital, Macau University of Science and Technology, Macau, Macao SAR, China; ^2^ Clinical Medical College, Southwest Medical University, Luzhou, China; ^3^ Biology Department, Southern Methodist University, Dallas, TX, United States; ^4^ Department of Radiation Oncology, National Cancer Center/National Clinical Research Center for Cancer/Cancer Hospital, Chinese Academy of Medical Sciences and Peking Union Medical College, Beijing, China; ^5^ Department of General Surgery, Dazhou Central Hospital, Dazhou, China; ^6^ Department of Specialty Medicine, Ohio University, Athens, OH, United States

**Keywords:** mitochondrial gene defects, pancreatic cancer, multiomics analysis, immune escape, disease progression, clinical application prospects

## Abstract

This comprehensive review delves into the complex interplay between mitochondrial gene defects and pancreatic cancer pathogenesis through a multiomics approach. By amalgamating data from genomic, transcriptomic, proteomic, and metabolomic studies, we dissected the mechanisms by which mitochondrial genetic variations dictate cancer progression. Emphasis has been placed on the roles of these genes in altering cellular metabolic processes, signal transduction pathways, and immune system interactions. We further explored how these findings could refine therapeutic interventions, with a particular focus on precision medicine applications. This analysis not only fills pivotal knowledge gaps about mitochondrial anomalies in pancreatic cancer but also paves the way for future investigations into personalized therapy options. This finding underscores the crucial nexus between mitochondrial genetics and oncological immunology, opening new avenues for targeted cancer treatment strategies.

## 1 Background

Pancreatic cancer, which is characterized by aggressive progression and a high mortality rate, is one of the most common fatal cancers worldwide (1, 2). Epidemiological studies indicate an increasing incidence of pancreatic cancer in industrialized nations, closely linked with aging demographics and lifestyle factors (3). The treatment landscape of pancreatic cancer is challenging and characterized by late-stage diagnosis and limited effective treatment options (2, 4). The ineffectiveness of conventional treatments is compounded by the prevalence of metastatic disease at diagnosis, coupled with a generally poor response to chemotherapy and radiation therapies (5, 6). Consequently, there is a pressing need for research focused on early detection methods and novel therapeutic approaches (7).

The role of mitochondrial gene defects has emerged as a significant area of interest in pancreatic cancer research (8, 9). These defects can lead to disrupted energy metabolism, increased oxidative stress, and changes in apoptotic pathways, all of which critically affect the survival and proliferation of pancreatic cancer cells (8, 10). Such metabolic and signaling disturbances not only facilitate tumor growth but also may contribute to resistance against standard therapies (11, 12). Furthermore, mitochondrial dysfunction is known to impact the tumor microenvironment, particularly affecting immune cell regulation, a key element in the immune escape mechanisms of pancreatic cancer (13, 14).

Employing a multiomics approach to study mitochondrial gene defects in pancreatic cancer offers significant insights (15). Through the integration of genomics, transcriptomics, and proteomics, we can comprehensively analyze the impact of these genetic defects on tumor cell metabolism, signaling, and immune responses (6, 16). This multifaceted analysis is crucial for understanding how mitochondrial gene defects influence the development of pancreatic cancer at the molecular level and for identifying new therapeutic targets, especially those that regulate immune responses and counteract immune evasion (17–20). As such, deepening our understanding of mitochondrial gene defects in pancreatic cancer is vital not only for deciphering disease mechanisms but also for advancing the development of innovative therapeutic strategies, notably in precision medicine and immunotherapy (18).

This review aims to provide an in-depth overview of the epidemiology, pathological features, and therapeutic challenges of pancreatic cancer, with an emphasis on the pivotal role of mitochondrial gene defects in its development. Additionally, we discuss the potential impact of these findings on the formulation of novel therapeutic strategies, particularly those focused on precision medicine and immunotherapy. Through this comprehensive analysis, we seek to offer new insights and directions for the research and treatment of pancreatic cancer.

## 2 Pancreatic cancer and mitochondrial function

### 2.1 Basic mitochondrial functions

Mitochondria, often referred to as cellular powerhouses, are critical for energy production within cells and play a central role in numerous cellular processes (21). They are primarily responsible for generating the bulk of cellular energy in the form of ATP through a process that involves a series of electron transport chain complexes. This process culminates in the activation of ATP synthase, which synthesizes ATP (22).

Furthermore, mitochondria are instrumental in regulating apoptosis, an orderly self-destructive process vital for maintaining tissue health. They exert this control primarily through the release of apoptosis-associated proteins, such as cytochrome. Once released into the cytoplasm, cytochrome c initiates a cascade of reactions culminating in cell death (23, 24).

In cancer cells, mitochondrial function often undergoes significant alterations. A notable example is the Warburg effect, where cancer cells preferentially generate energy through glycolysis rather than oxidative phosphorylation, even in oxygen-rich conditions. This metabolic shift plays a crucial role in the rapid growth and survival of cancer cells (25–28). Specifically, in pancreatic cancer, such mitochondrial metabolic reprogramming is closely linked to increased tumor aggressiveness, resistance to treatment, and a poorer prognosis (29).

### 2.2 Role of mitochondria in tumor progression

**Figure 1:**
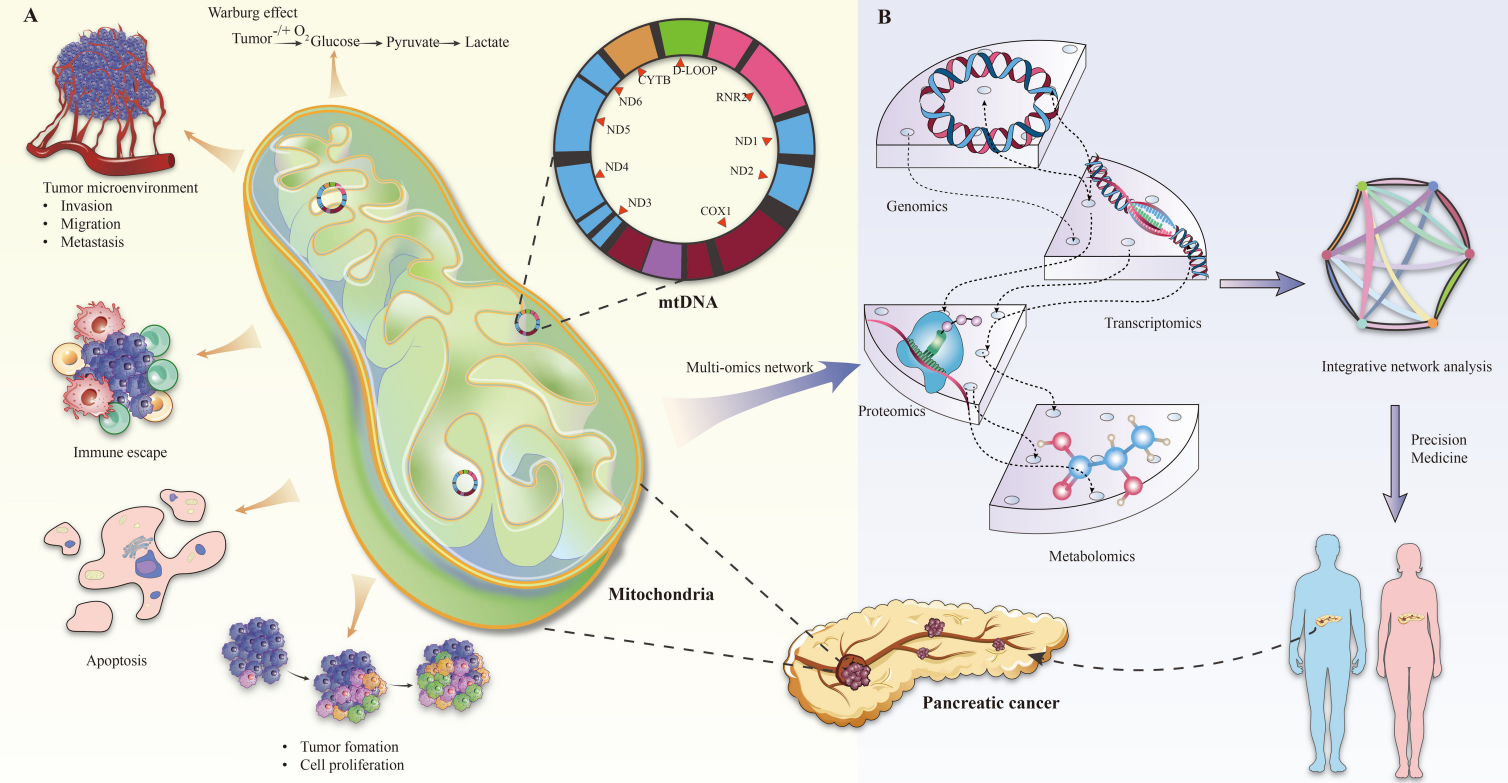
**(A)** Mitochondrial changes in pancreatic cancer. **(B)** Multi-omics analysis of mitochondrial gene defects in pancreatic cancer.

The role of mitochondria in tumor progression manifests primarily in three areas: metabolic reprogramming, anti-apoptotic mechanisms, and immune escape, all of which are crucial for cancer progression and resistance to therapy (13, 30–32) (**Figure 1A**).

The Warburg effect is important for tumor metabolic reprogramming. The (platelet and lymphocyte ratio) PLR describes the propensity of cancer cells to rely on glycolysis for energy production, even in the presence of adequate oxygen (33–35). Although ATP production is less efficient than oxidative phosphorylation, glycolysis supplies rapidly dividing cancer cells with necessary biosynthetic precursors. In pancreatic cancer, for instance, this metabolic shift is facilitated by the upregulation of key glycolytic enzymes such as hexokinase and lactate dehydrogenase, promoting tumor growth and contributing to alterations in the tumor microenvironment. This includes environmental acidification, which subsequently impacts the interactions between tumors and immune cells (29, 36).

The role of mitochondria in antiapoptotic mechanisms is equally significant. Cancer cells often evade apoptosis by modulating mitochondrial pathways, thus ensuring their survival and proliferation (37, 38). This evasion is largely orchestrated through the regulation of the Bcl-2 protein family, which comprises both proapoptotic (e.g., Bax and Bak) and antiapoptotic (e.g., Bcl-2 and Bcl-xl) members (39–44). In various cancers, such as certain breast cancers and leukemias, resistance to apoptosis is achieved by either upregulating antiapoptotic proteins (e.g., Bcl-2) or suppressing proapoptotic proteins (e.g., Bax) (41, 45–47). For instance, overexpression of Bcl-2 in tumor cells has been linked to resistance against certain chemotherapeutic agents (48–50).

In addition, mitochondrial dysfunction is increasingly recognized as a contributing factor to immune escape mechanisms in the tumor microenvironment (30, 51). Mitochondria influence the tumor microenvironment by regulating inflammatory responses and cytokine production, which in turn affects immune cell infiltration and activity (13, 31). Therefore, in some tumors, the anti-tumor immune response is weakened due to impaired mitochondrial function, providing favorable conditions for tumor cell escape and proliferation (30).

## 3 Multiomics analysis of mitochondrial gene defects and pancreatic cancer

In the field of pancreatic cancer research, multiomics analyses have played a pivotal role in elucidating the impact of mitochondrial gene defects. Alterations in the mitochondrial genome (mtDNA), such as mutations or deletions, have been associated with increased chemoresistance and metastatic capabilities in various cancer types. Moreover, recent studies have highlighted the potential of microRNAs that regulate mtDNA-encoded mitochondrial proteins (mitomiRs) and nuclear-encoded mitochondrial proteins as valuable biomarkers for cancer diagnosis and prognosis (9). For example, in the diagnosis of pancreatic cancer, several miRNAs (pancreatic intraepithelial neoplasia) have already been identified by researchers in the PanIN (pancreatic intraepithelial neoplasia) staging of pancreatic cancer, allowing us to study them as potential biomarkers (52).

Genomic analysis plays a crucial role in identifying specific mutations in the mitochondrial DNA of pancreatic cancer cells. An example is the point mutation in the MT-ND4 gene, which is known to disrupt the mitochondrial electron transport chain, leading to functional deficits (53, 54).

Transcriptomic analyses complement these findings by shedding light on the impact of these genetic variations on mitochondrial gene expression. In some cases, pancreatic cancer cells exhibit altered expression of mitochondria-encoded subunits of the oxidative phosphorylation complex, directly influencing cellular energy metabolism (17).

Proteomics studies have delved into the detection of aberrant expression of mitochondrial proteins in cancer cells by mass spectrometry to further confirm that changes in mitochondrial protein function may lead to unexpected mitochondrial dysfunction that can cause disease. This technique, particularly quantitative mitochondrial proteomics, provides a thorough and precise analysis of mitochondrial protein levels, including posttranslational modifications (PTMs), thus offering a comprehensive view of the changes in mitochondrial protein dynamics in cancer cells (55). For example, a study analyzing colorectal cancer tissue revealed significant alterations in the expression of mitochondrial enzymes involved in the Krebs cycle, including a marked increase in the levels of malate dehydrogenase, a key enzyme in this pathway (56). In pancreatic cancer, similar studies have linked changes in the levels of mitochondrial respiratory chain complexes I and II to increased oxidative stress (57).

Metabolomic analyses contribute to elucidating the changes in the metabolic profiles of pancreatic cancer cells caused by mitochondrial gene defects (58, 59). Notably, the increased production of lactate signals a shift toward glycolysis, which is a hallmark of cancer cell metabolism (17, 60).

Overall, the integration of genomic, transcriptomic, proteomic, and metabolomic analyses provides a holistic understanding of the role of mitochondrial gene defects in pancreatic cancer development (**Figure 1B**). For example, Rae-Anne Hardie and her team performed a multiomics analysis of mitochondrial DNA from 12 pancreatic cancer cell lines (PDCL) and identified 24 somatic mutations in them. The study showed metabolic changes consistent with mitochondrial dysfunction, including decreased oxygen consumption and increased glycolysis (17). Furthermore, individual tumors adapt to increased anabolic demands through different genetic mechanisms, so targeting therapy to the resulting metabolic phenotype may be an effective strategy. This multifaceted approach not only enhances our understanding of disease pathogenesis, but also holds promise in guiding the development of future therapeutic strategies, especially in the context of personalized medicine.

## 4 Mitochondrial gene defects and pancreatic cancer development

### 4.1 Specific genetic defects

In the context of pancreatic cancer, particular mitochondrial gene defects significantly influence tumor biology (17, 18, 61, 62). Mutations in genes such as MT-ND4 (mitochondrial NADH dehydrogenase subunit 4) have been implicated in compromising the mitochondrial electron transport chain, leading to increased intracellular oxidative stress and destabilized energy metabolism (54, 63). The specific study found that the majority of mtDNA mutations (41.5%) in PDAC-EV were located in respiratory complex I (RCI) (ND1-ND6), followed by the RCIII gene (CYTB; 11.2%).This provides a solid foundation for further research into mtDNA biomarkers for PDAC diagnosis and the development of innovative, clinically feasible EV-based assays. Additionally, variations in mitochondrial DNA copy number, particularly in the MT-CO1 gene (mitochondrial cytochrome c oxidase subunit I), are linked with the proliferative and invasive capacities of pancreatic cancer cells (64, 65). These genetic anomalies not only affect the metabolic and survival pathways of tumor cells but also substantially impact the tumor microenvironment, consequently influencing the progression of cancer and the response to therapy. Thus, understanding these mitochondrial gene defects is vital for comprehending the pathogenesis of pancreatic cancer and for developing new therapeutic strategies (**Table 1**).

**Table 1:** Mitochondrial gene defects in pancreatic cancer.

Analysis region	Analysis type	Samples	Mutation	Findings and conclusions	Reference
mtDNA in the EVs	mtDNA content	Serum samples; pancreatic cancer patients (n=20), non-cancer subjects (n=10)	Mainly in RCI (ND1-ND6), RCIII genes (CYTB), D-Loop, RNR2	Circulating EVs detects mtDNA mutations and may diagnose PDAC	(66)
Whole mtDNA	mtDNA copy number	Peripheral blood leukocyte samples; PDAC cases (n=476), controls (n=357)		Increased mtDNA copy number is associated with reduced risk of developing PDAC	(66)
Whole mtDNA	mtDNA copy number	Tissue samples; resectable pancreatic ductal adenocarcinoma (n = 43), adjacent normal pancreas (n = 31)		Mitochondrial DNA copy number is significantly lower in pancreatic cancer tissue and is not a significant predictor of prognosis in resectable pancreatic cancer	(67)
Whole mtDNA	mtDNA copy number	268 mitochondrial genomes from early resected pancreatic ductal adenocarcinoma tissues and paired non-tumor tissues	Frequent mutations in ND5, RNR2, CO1, transfer RNA genes (n=29)	61% of the tumor samples had at least 1 mtSNV; The mtSNVs in ND4 and ND6 were associated with shorter overall survival times; Tumors accumulate mitochondrial mutations with progression	(68)
Whole mtDNA	Mutations	12 patient-derived pancreatic cancer cell lines (PDCL)	24 somatic mutations in the mtDNA (ETC complex I subunit (n = 9), noncoding control region (n = 10), COI of complex IV (n = 2)), CyB of complex III (n = 3))	Decreased oxygen consumption and increased glycolysis consistent with mitochondrial dysfunction; Heterogeneous genomic landscapes of pancreatic tumors may converge on common metabolic phenotypes	(16)

RCI, respiratory complex I; PDAC, pancreatic ductal adenocarcinoma; mtSNV, mitochondrial single nucleotide variant (defined as a location where the difference in heterogeneity score between tumor and normal samples is ≥0.2); COI, cytochrome C oxidase subunit I; CyB, cytochrome B0.

### 4.2 Metabolism and the tumor microenvironment

Mitochondrial gene defects profoundly alter the metabolic landscape of the pancreatic cancer tumor microenvironment (13, 18). These defects typically result in enhanced glycolysis and reduced oxidative phosphorylation, culminating in acidification of the tumor microenvironment (66, 69, 70). This acidic milieu not only promotes the survival and proliferation of tumor cells but also hinders the functioning of immune cells, thus facilitating tumor immune escape. Furthermore, mitochondrial dysfunction is associated with elevated reactive oxygen species production, which contributes to DNA damage, gene mutation, and accelerated tumor progression (71–73). Therefore, mitochondrial metabolism is a crucial factor in shaping the tumor microenvironment, impacting both tumor growth and the effectiveness of therapeutic interventions.

## 5 Mitochondrial gene defects from an immunologic perspective

### 5.1 Mitochondrial gene defects and immune escape

Mitochondrial gene defects significantly contribute to immune escape mechanisms in pancreatic cancer (13). Mutations in genes such as MT-CO1 and MT-ND4 can lead to dysfunction in the mitochondrial electron transport chain, affecting oxidative stress levels and altering metabolite concentrations within cells (64, 74, 75). These changes may disrupt antigen processing and presentation on the cell surface, thus impairing the recognition of tumor cells by immune cells. For instance, mitochondrial defects that alter metabolites such as lactate can acidify the tumor microenvironment, which suppresses T-cell activity and supports tumor immune escape (76–79).

### 5.2 Regulation of the immune microenvironment

Mitochondrial functionality is pivotal in regulating the immune microenvironment in pancreatic cancer (80). Through their influence on metabolic pathways, including ROS production and energy metabolism, mitochondria indirectly regulate the infiltration and activity of immune cells. Increased ROS production triggers the release of inflammatory factors such as IL-6 and TNF-α, altering the tumor microenvironment, which in turn affects the function of T cells and other immune cells (81–83). In addition, mitochondrial adenosine triphosphate (ATP) synthase, which produces most of the ATP required by mammalian cells, has been shown to decrease ATP production when cellular respiration is impaired, potentially affecting autophagy and immune function (84, 85). In certain pancreatic cancer models, the inflammatory milieu resulting from mitochondrial dysfunction has been shown to promote the recruitment of immunosuppressive cells, such as tumor-associated macrophages, further enhancing the tumor’s ability to evade the immune response (18, 86, 87).

## 6 Clinical application and therapeutic prospects

The clinical application and therapeutic prospects of pancreatic cancer treatment are increasingly focused on two main areas: the development of targeted therapeutic strategies and the implementation of precision medicine (88, 89). In regard to therapeutic strategies, a key area of exploration is the creation of drugs that specifically target mitochondrial gene defects in pancreatic cancer. For instance, targeted therapies aimed at identifying mutations within the MT-CO1 gene could inhibit tumor growth by restoring normal mitochondrial electron transport chain function. Additionally, the use of metabolic modulators such as 2-deoxyglucose (2-DG) might prove especially effective in targeting pancreatic cancer cells exhibiting the Warburg effect by impeding their glycolytic pathways (18, 29). In the realm of precision medicine, the integration of multiomics data—including genomics, transcriptomics, and proteomics—enables the development of personalized treatment plans for patients with pancreatic cancer (90–92). This approach involves analyzing the mitochondrial genome of a patient’s tumor to identify and tailor therapies that are best suited to the patient’s unique genetic profile. For instance, selecting small molecule inhibitors that specifically target identified mitochondrial mutations can be a direct outcome of this analysis (93). This method also has the potential to better predict a patient’s response to standard chemotherapy, thereby optimizing treatment regimens, reducing adverse effects, and improving overall treatment efficacy.

## 7 Discussion

Mitochondria are important organelles responsible for many physiological processes, such as energy production, apoptosis, and cellular metabolism. Mitochondrial dysfunction is increasingly recognized as a central mediator of many common diseases, including tumors and cardiovascular diseases, and there is growing evidence that mitochondrial metabolic disorders are involved in cancer development, which explains the significance of the Warburg effect for metabolic reprogramming in tumors (94). Furthermore, specific mitochondrial gene defects substantially affect the tumor microenvironment by influencing metabolic pathways, (including ROS production and energy metabolism), enhancing immune escape of cancer cells; mutations alter cytokine and chemotactic factor release, thereby affecting immune cell infiltration and function in the tumor microenvironment (95). For instance, mitochondrial dysfunction may foster the recruitment of immunosuppressive cells such as tumor-associated macrophages, subsequently dampening T-cell-mediated immune responses (96).

A key future research trajectory is the development of targeted therapies for specific mitochondrial gene mutations. Employing small molecule drugs or gene editing techniques to correct these genetic anomalies could restore mitochondrial function and impede tumor growth. Additionally, the use of metabolic modulators may augment the efficacy of chemotherapy and immunotherapy (97).

The advancement of personalized treatment strategies represents a significant research avenue (98). The integration of multiomics data enables the formulation of optimal treatment plans tailored to the unique genetic and microenvironmental characteristics of each patient’s tumor (99–101). This personalized approach holds the promise of a major breakthrough in pancreatic cancer treatment, potentially enhancing patient prognosis and survival.

Interestingly, we also found that mitochondrial retrograde signaling is also important for metabolic activities (13). Mitochondrial retrograde signaling communicates with the nucleus to maintain mitochondrial homeostasis and respond to stress. This process involves mitochondria-derived molecules (ROS, calcium, exported mtDNA, mitochondrial double-stranded RNA), the mitochondrial unfolded protein response (mtUPR), and the integrative stress response (ISR). Among these, ROS is a by-product of mitochondrial respiration and an important mediator of retrograde mitochondrial signaling. These findings suggest that targeting mitochondrial retrograde signaling may be a potential therapy against cancer progression. In addition, it has been found that mtDNA mutations can be detected in circulating extracellular vesicles (EVs) and may serve as a reliable diagnostic tool for pancreatic ductal adenocarcinoma (PDAC), compensating for the lack of highly sensitive and specific biomarkers for the diagnosis of early-stage pancreatic ductal adenocarcinomas and enriching the choice of multimodal therapy (18).

## Author contributions

HC: Conceptualization, Data curation, Validation, Writing – original draft, Writing – review & editing. LS: Data curation, Writing – original draft. YY: Data curation, Writing – original draft. XG: Writing – original draft. KS: Writing – original draft. HL: Writing – original draft. LY: Writing – original draft. JL: Writing – original draft. JW: Writing – original draft, Writing – review & editing. QW: Writing – original draft, Writing – review & editing. GY: Writing – original draft, Writing – review & editing.
